# Health-related Information on the Web: Results From the HealthStyles Survey, 2002–2003

**Published:** 2006-03-15

**Authors:** Pooja Bansil, Nora L Keenan, Amy I Zlot, Jeanne C Gilliland

**Affiliations:** National Center for Chronic Disease Prevention and Health Promotion, Centers for Disease Control and Prevention; Cardiovascular Health Branch, National Center for Chronic Disease Prevention and Health Promotion (NCCDPHP), Centers for Disease Control and Prevention (CDC), Atlanta, Ga; Office of Informatics and Information Resource Management, NCCDPHP, CDC, Atlanta, Ga; Office of Informatics and Information Resource Management, NCCDPHP, CDC, Atlanta, Ga

## Abstract

**Introduction:**

The World Wide Web is being used increasingly as a resource for accessing health-related information. In our study, we identified types of health-related Web sites visited most often, determined how often patients shared Web-accessed health information with their doctors, and examined factors that encouraged Internet use for locating health-related information. We also compared health-related Internet use among people who did not have any type of chronic disease with people who reported having one or more chronic diseases.

**Methods:**

We merged data from the 2002 and 2003 HealthStyles surveys to generate frequency and descriptive statistics and used multivariate logistic regression to estimate odds ratios.

**Results:**

Approximately 35% of survey participants reported using the Internet to search for health-related information. Among them, the Web sites visited most often included health information portals, government agencies, and nonprofit organizations. About 53% reported that they "sometimes" shared Internet information with their doctors. The most important features of the Internet that would encourage its use for health information were ease of finding and using the information and clarity of the information provided. Internet use differed by sex and age and was strongly associated with income and education. Respondents who reported having a chronic disease (odds ratio [OR] = 1.30; 95% confidence interval [CI], 1.16–1.45) were more likely to use the Internet to access health-related information, especially among those with depression (OR = 1.47; 95% CI, 1.27–1.71) and high cholesterol (OR = 1.18; 95% CI, 1.02–1.37). In addition, respondents who reported having two or more chronic diseases (OR = 1.35; 95% CI, 1.16–1.56) were more likely to search for online health information than respondents who reported having no chronic disease.

**Conclusion:**

Public health professionals have a unique opportunity to use the Internet as a tool to complement and supplement the health information that the public receives from health care professionals.

## Introduction

More and more people are using the Internet to access health-related information. In 2003, an estimated 93 million Americans used the Internet for this purpose, a 27% increase over 2002 (1,2). People use the Internet because of several favorable features: the convenience of being able to search quickly for information at any time, unlimited access to inexpensive information resources, and user anonymity when searching for subject-sensitive health information (1,3-5).

Several studies have found that the health topics most frequently searched on the Internet include the following: 1) background information as well as symptoms and treatment of a disease or condition, 2) medications and prescription drugs, 3) diagnostic tools, 4) new or experimental treatments, 5) diet, 6) exercise, and 7) support groups (2,6-9). Although 86% of Internet users indicated concern about obtaining unreliable information, 52% of participants who used health sites thought that "almost all" or "most" health information found on the Internet was credible (1). In contrast, 84% of physicians rate their patients as only "fair or poor" at appraising the quality of Web-accessed information and believe that although accurate or relevant information can be beneficial, inaccurate or irrelevant information can be harmful to patients' health care and outcomes (10). Results from a systematic review, however, found a low risk of harm associated with using the Internet as a health information resource (11).

Because of the wide range in quality of health information available on the Internet, one major concern is where people search for health-related information on the Internet. In addition, having access to numerous inexpensive sources that may or may not be credible raises questions about whether people who search the Internet for health information also seek advice from their physicians when experiencing serious health problems (12). Using data from a recent mail survey of the U.S. population, we characterized the population that uses the Internet for accessing health information, and we evaluated use of various types of health-related Web sites. We also explored how often individuals in this population shared health-related information found on the Internet with their physicians. Because the Internet can be used as a valuable tool for both health promotion and disease prevention, we examined factors that would encourage using the Internet to search for health information.

Because 67% of all deaths are attributable to five chronic diseases (heart disease, cancer, stroke, chronic obstructive pulmonary disease, and diabetes) and because these diseases account for 75% of the nation's total health care cost (13), we examined whether using the Internet as a health information resource differed among individuals having one or more chronic diseases compared with those having no chronic disease.

## Methods

### Study design 

We selected our study population from respondents to the 2002 and 2003 HealthStyles surveys. The HealthStyles survey, developed by Porter Novelli, a social marketing and public relations firm, with technical assistance from the Centers for Disease Control and Prevention, is one of a pair of linked postal mail surveys and is designed to collect data on health attitudes, behaviors, conditions, and knowledge (14). A stratified random sample is generated based on age, sex, marital status, race and ethnicity, income, geographic region, household size, and population density. In addition, low-income and minority households are oversampled to improve their representation in the sample (14,15). The HealthStyles survey is sent as a follow-up to the ConsumerStyles survey, which is administered annually to approximately 10,000 potential respondents. A total of 6027 HealthStyles surveys in 2002 and 5845 HealthStyles surveys in 2003 were mailed to selected respondents. Responses were received from 4397 (73%) participants in 2002 and from 4035 (69%) participants in 2003, yielding a total of 8432 eligible study participants.

### Study variables

The 2002 and 2003 HealthStyles surveys included a series of questions about participants' use of the Internet for accessing health-related information. Respondents were first asked a screening question, "Do you look for health information on the Web?" If they answered yes, they were asked additional questions about their use of the Web for accessing health-related information; if they answered no, they skipped to the next section of the survey. Another question asked of those who reported seeking health-related information on the Web was, "When looking for health information on the Web, which Web sites do you frequently visit?" Categories included 1) governmental agencies, 2) nonprofit organizations, 3) health information portals, 4) scientific retrieval systems, 5) universities, 6) health chat rooms, 7) physicians' offices, hospitals, or health centers, 8) online newsletters, and 9) pharmaceutical companies. Respondents were also asked how often (always, usually, sometimes, never) they discussed the health information that they found on the Web with their physicians and about possible features of the Internet that would encourage them to search for online health-related information.

Other questions of interest included self-reported health status (excellent, very good, good, fair, poor) and health conditions. We dichotomized health status into excellent/very good/good and poor/fair and classified respondents as having a chronic disease if they reported having one of the following chronic conditions included on the HealthStyles survey: arthritis, asthma, cancer, diabetes, high blood pressure, high cholesterol, osteoporosis, or depression. Because the respondents could report more than one chronic condition, we further classified each participant into one of three groups based on the number of chronic conditions that they reported (none, one, or two or more).

### Statistical analyses 

HealthStyles data from 2002 and 2003 were merged, and the entire sample was weighted for sex, age, race, income, and household size to represent the U.S. census population. Frequency analyses provided information on the percentage of Internet users who conducted health information searches. Descriptive statistics of sociodemographic variables (sex, age, race and ethnicity, marital status, education, and income) were used to characterize members of the sample population who reported using the Internet as a health information resource and to compare them with members who reported not using the Internet for such information. Multivariate logistic regression was used to investigate the relationship between having a chronic disease and use of Web as a health information resource. In addition, we assessed associations between selected chronic diseases and the collective number of chronic diseases and the use of the Internet to seek health-related information. We further categorized age (18–34 years, 35–54 years, and 55 years and older) and annual income (<$25,000, $25,000–59,999, and ≥$60,000). Respondents who reported race as "other" were excluded from the analyses. A stepwise backward elimination approach was used to select covariates for the final model; α was set at .05. All analyses were performed using SAS version 9.1 (SAS Institute Inc, Cary, NC).

## Results

Of the total 8432 eligible HealthStyles respondents in 2002 and 2003, 52% were women and 48% were men. The mean age was 48 years, and 22% were aged between 35 and 44 years. Most respondents were white (72%) and were married (59%); approximately one third had some college education (32%) and earned $60,000 or more per year (32%). Sixty-nine percent reported having good health or better, and 59% had one or more chronic diseases.

Thirty-nine percent of the women and 33% of the men used the Web as a health information resource (Table 1). On average, use of the Web for seeking health information was highest among respondents aged 25 to 34 years (43.5%), Asians or Pacific Islanders (49.2%), and those who were married (37.6%). Seeking health-related information on the Web increased with level of education (*P* < .001) and income (*P* < .001). Participants who reported their general health as being fair or poor sought online health information more often than participants who reported their health as being excellent, very good, or good.

**Figure 1:**
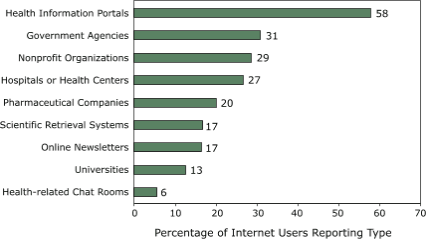
Types of Web sites frequently visited by survey respondents who reported using the Internet to seek health-related information (n = 2966) and percentage of respondents who reported using the type. Source: HealthStyles survey data from 2002 and 2003.

**Figure 2:**
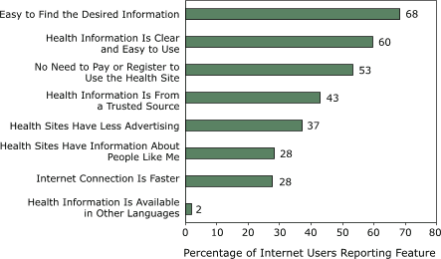
Features of the Internet that would encourage using the Web as a source for health-related information and the percentage of respondents (n = 2966) who reported them. Source: HealthStyles survey data from 2002 and 2003.

We included 2966 (35%) respondents who reported seeking health information on the Internet in a subgroup analysis. Among these respondents, health information portals were the most frequently used Internet source for health information (Figure 1); 58% of respondents reported using health information portals, 31% reported using government agency sites, and 29% reported using nonprofit organization sites. When respondents were asked which features of the Internet encouraged them to search for online health-related information (Figure 2), 68% reported ease of finding the desired information as the primary factor. Other factors included clarity and ease of use of accessed information (60%), not having to pay or register to use the Web-based health site (53%), and trustworthiness of the information source (43%). Among respondents who reported accessing health information on the Web, 53% reported sharing the information with their physicians sometimes; 11%, usually; 3%, always; and 32%, never (data not shown).

**Table:** 

**Type of Web Site Visited**	**% of Internet Users Reporting Type**
Health-related chat rooms	6
Universities	13
Online newsletters	17
Scientific retrieval systems	17
Pharmaceutical companies	20
Hospitals or health centers	27
Nonprofit organizations	29
Government agencies	31
Health information portals	58

**Table:** 

**Feature of Internet**	**% of Internet Users Reporting Feature**
Health information is available in other languages	2
Internet connection is faster	28
Health sites have information about people like me	28
Health sites have less advertising	37
Health information is from a trusted source	43
No need to pay or register to use the health site	53
Health information is clear and easy to use	60
Easy to find the desired information	68

Of the respondents who reported seeking health information on the Internet, 57% reported having one or more chronic diseases. Of these respondents, 37% reported having high blood pressure; 33%, high cholesterol; 33%, arthritis; 31%, depression; 18%, asthma; 16%, diabetes; 5%, osteoporosis; and 3%, cancer. Internet users who had one or more chronic conditions differed significantly by age (*P* < .001), race (*P* < .001), income *(P* = .009), education level *(P* < .001), marital status *(P* = .006), and health status *(P* = .02), but they did not differ significantly by sex (data not shown). Respondents with no chronic diseases differed significantly in their use of type of Web site from those who reported having one or more chronic diseases only in the use of health-related chat rooms (*P* = .002). Similarly, significant differences were found in the following features of the Internet that would encourage use of the Web more often to find health-related information: 1) easy to find the desired information (*P* < .001), 2) health sites have less advertising (*P* = .01), 3) Internet connection is faster (*P* = .01), and 4) health sites have information about people like me (*P* < .001) (data not shown).

After adjusting for sex, age, education, and income, results from multivariate logistic regression indicated that women were 1.36 (95% confidence interval [CI], 1.21–1.52) times as likely as men to use the Internet as a resource for health information (Table 2). People with at least a college degree were 2.68 (95% CI, 2.30–3.13) times as likely to seek health information online as people with a high school degree or less. In addition, respondents who earned $60,000 or more per year were twice as likely as respondents who earned less than $25,000 (odds ratio [OR] = 2.10; 95% CI, 1.78–2.48) to seek health information online. The odds of searching the Internet for health information decreased with increasing age; no significant effect of race on Internet use was found.

Internet users with one or more reported chronic disease were 1.30 (95% CI, 1.16–1.45) times as likely to seek health-related information as Internet users with no chronic disease. Furthermore, respondents with depression (OR = 1.47; 95% CI, 1.27–1.71) and high cholesterol (OR = 1.18; 95% CI, 1.02–1.37) were as likely to seek health-related information on the Web compared with those without these conditions. From regression analyses on number of chronic diseases, in contrast to the unadjusted analyses, respondents who reported having one chronic disease were 1.26 (95% CI, 1.11–1.44) times as likely to seek health information on the Web and respondents who reported having two or more chronic diseases were 1.35 (95% CI, 1.16–1.56) times as likely as respondents who reported having no chronic diseases.

## Discussion

Thirty-five percent of our study population used the Internet to search for health-related information. In contrast, results from the Health Information National Trends Survey (HINTS), the purpose of which is to identify the health information needs and practices among the American public (16), showed that 53% of respondents used the Internet to search for health or medical information for themselves and 48% searched for someone else (17). This difference may be explained by distinct differences in study design between the HealthStyles survey and HINTS.  

Internet use differed according to sex and age and was strongly associated with income and education. Women were more likely than men to seek health information, perhaps because women are more often considered caretakers and thus may be more concerned with their own health as well as the health of their family and friends. Although older people (aged 65 or older) are shown to be the fastest growing age group of online users (18), the activity of searching the Internet for health information decreased with age in our study. Our finding could possibly be attributed to the fact that older people are more likely than younger people to be new Internet users and thus often lack familiarity with computers and have limited knowledge of how to conduct and interpret Internet searches (18). One study found that 77% of Internet users who had 2 or 3 years of online experience searched for health information on the Web and that 59% of new Internet users made such searches (2). Similarly, previous use of health-related Web sites was found to be a significant predictor of interest in online health information (19). 

Although Internet access is strongly associated with income and education, differences in Internet use for health-related information cannot be solely attributed to lack of Internet access (8,18). In a study in which participants received free Internet access, only one fourth of them actually searched the Internet for health information (20). Consequently, differences in Internet use also may reflect other priorities, more compelling needs, and lack of interest in health information as well as education, income, and content barriers such as literacy, language, and cultural diversity (8,18,21,22). 

As previously stated, respondents with either one or two or more chronic conditions were more likely than respondents with no chronic conditions to search for online health-related information. This finding could be attributed to the possibility that people with chronic health conditions want to control and manage their illnesses. Several studies have shown that using the Internet to obtain health-related information improved people's understanding of a disease or condition, increased their knowledge of treatment options and outcomes, influenced their exercise or eating behavior, influenced their treatment decision making, and helped in coping with their illness (5,6,8,10,12,22,23). In addition, 85% of physicians interviewed reported that patients experienced benefits from using the Internet to search for health-related information (22). Many health care providers also believe that patients who are well educated about health are more involved in their own health care and adhere better to treatment regimens (5,10,23-26). A significant difference in the use of health-related chat rooms among people with one or more chronic diseases compared with those who have none could be because chat rooms not only allow patients to exchange information and provide emotional support but also promote disease management and adherence to medication (27).

Although using the Internet did not affect the number of visits or phone calls patients made to their physicians (6), the majority (53%) of our study sample sometimes shared information that they accessed on the Web with their doctors. Possible reasons why patients might hold back information included feeling uncomfortable discussing highly personal issues, having less time to talk with their physician, and fear of receiving negative feedback from their physician (5,22). Nonetheless, in a survey of doctors' experiences of patients using the Internet, 44% of the physicians reported that their patients experienced problems in using the information obtained from the Internet, and 8% reported that their patients experienced actual physical harm (22). The most commonly reported problems included obtaining misinformation about a condition and patient requests for new, unavailable treatments and alternate or experimental therapies (5,22). Hence, it is imperative that patients be encouraged to always share concerns, including health-related information that they access from the Internet, with their doctors to avoid adverse health outcomes.

In this study, the three types of Web sites (i.e., health information portals, government agencies, and nonprofit organizations) most frequently cited by Internet users for researching health-related information indicate that the public is accessing high-quality, credible online health information from trustworthy sources (28). Web sites from health-related government agencies, academic health science centers, and commercial health information publishers are likely to have high-quality information (25). Web sites ending in ".com" may have self-interests that compromise the quality or completeness of the information presented compared with Web sites ending in ".org," ".edu," or ".gov" (26).

The two most important factors that would encourage Internet users to use the Web more often as a health information resource are ease of finding the desired information and health information that is clear and easy to use. Hence, Web sites should present information clearly and should preferably be written at a seventh- or eighth-grade reading level so that all Internet users can use the information as a tool to improve their health. It is also important that 37% of respondents reported that having fewer advertisements would also encourage them to use a Web site. Advertisements are viewed as a red flag, indicating that the information might be biased toward marketing partners (28).

The findings reported in this study may be subject to several limitations. First, because HealthStyles is a mail survey, the sampling methods used generate a sample population in which minority and low-income households are underrepresented. Nonetheless, the HealthStyles survey is based on a national sample, and its data correlate well with surveillance data from the Behavior Risk Factor Surveillance System (14). Second, we relied on self-reported data, which might not provide complete or accurate findings on Internet use for obtaining health-related information.

Facilitating appropriate use of the Internet to locate and interpret high-quality health information is a worthy challenge for the future. Public health professionals and the medical community can play key roles in this endeavor by becoming more aware of the types of information available on the Internet, by critically appraising the available information, by rating the value and trustworthiness of the sources, and by helping to identify high-quality sites. In addition, health care providers should consider opportunities for using the Internet to communicate with their patients. Results from the HINTS survey indicate that 7.6% of respondents reported using e-mail to communicate with a physician or physician's office (17). Furthermore, studies on the use of e-mail and Web messaging as tools for communication between patients and their physicians have shown that these were easy to use, allowed flexibility in communicating nonurgent problems, improved continuity of care overall, and enhanced disease management (29,30).

In recognizing the increasingly important role that the Internet plays in our nation's health, both the public health and medical communities have a unique opportunity to use it as a valuable tool for health promotion and disease prevention. The Internet can help educate patients, enrich the patient–physician relationship, and influence patients' health care and health outcomes. We encourage further studies that evaluate physicians' reactions to patients searching for online health information, explore additional barriers that the public faces when searching for online health information, assess how successful people are in finding health information, and determine how people use the information (i.e., diagnosis, treatment, or prevention).

## Figures and Tables

**Table 1:** Demographic Characteristics of Survey Participants According to Whether They Seek Health-related Information on the Web, HealthStyles Surveys, 2002–2003

**Characteristic**	**Yes (n = 2966) No. (%)**	**No (n = 5354) No. (%)**	***P* Value**

**Sex**			

Male	1299 (32.5)	2702 (67.5)	<.001
Female	1667 (38.6)	2562 (61.4)	

**Age, y**			

18-24	476 (43.2)	625 (56.3)	<.001
25-34	667 (43.5)	865 (56.5)	
35-44	713 (39.3)	1099 (60.7)	
45-54	612 (39.3)	947 (60.7)	
55-64	301 (30.3)	693 (69.7)	
≥65	196 (14.9)	1125 (85.1)	

**Race or ethnicity**			

White	2162 (35.9)	3857 (64.1)	<.001
Black	306 (31.8)	658 (68.2)	
Hispanic	308 (33.3)	619 (66.7)	
Asian or Pacific Islander	154 (49.2)	159 (50.8)	
Other	35 (36.6)	61 (63.4)	

**Marital status**			

Married	1856 (37.6)	3087 (62.4)	<.001
Not marrieda^a^	1058 (32.8)	2167 (67.2)	

**Education**			

High school or less	471 (21.4)	1734 (78.6)	<.001
Some college	1055 (39.9)	1589 (60.1)	
College degree or more	1120 (47.1)	1261 (52.9)	

**Household income, $**			

<15,000	272 (23.0)	910 (77.0)	<.001
15,000-24,999	290 (25.8)	837 (74.2)	
25,000-39,999	491 (32.0)	1045 (68.0)	
40,000-59,999	577 (37.7)	952 (62.3)	
≥60,000	1223 (45.8)	1446 (54.2)	

**Health status**			

Excellent/very good/good	1987 (34.9)	3716 (65.1)	.02
Poor/fair	962 (37.5)	1601 (62.5)	

**Chronic diseases**			

Yes	1681 (34.3)	3216 (65.7)	.003
No	1285 (37.5)	2137 (62.5)	

a Includes divorced, widowed, separated, and never married.

**Table 2:** Likelihood of Seeking Health-related Information on the Web, by Sociodemographic Characteristics, HealthStyles Surveys, 2002–2003

**Characteristic**	**UnadjustedOdds Ratio (95% Confidence Interval)**	**Adjusted^a^ Odds Ratio (95% Confidence Interval)**

**Sex**		

Male	1.00 (Referent)	1.00 (Referent)
Female	1.31 (1.20-1.43)	1.36 (1.21-1.52)

**Age, y**		

18-34	1.00 (Referent)	1.00 (Referent)
35-54	0.85 (0.76-0.94)	0.67 (0.59-0.76)
≥55	0.56 (0.48-0.66)	0.43 (0.36-0.52)

**Education level**		

High school or less	1.00 (Referent)	1.00 (Referent)
Some college	2.45 (2.15-2.77)	2.03 (1.76-2.34)
College degree or more	3.25 (2.86-3.71)	2.68 (2.30-3.13)

**Household income, $**		

<25,000	1.00 (Referent)	1.00 (Referent)
25,000-59,999	1.68 (1.49-1.90)	1.57 (1.35-1.83)
≥60,000	2.68 (2.37-3.03)	2.10 (1.78-2.48)

**Chronic disease**		

No	1.00 (Referent)	1.00 (Referent)
Yes	0.87 (0.79-0.95)	1.30 (1.16-1.45)

**Type of chronic disease**		

None	1.00 (Referent)	1.00 (Referent)
Asthma	1.15 (0.99-1.34)	1.19 (0.99-1.44)
Arthritis	0.63 (0.57-0.71)	1.10 (0.94-1.27)
Cancer	0.83 (0.59-1.19)	1.30 (0.75-2.24)
Depression	1.37 (1.21-1.55)	1.47 (1.27-1.71)
Diabetes	0.75 (0.64-0.87)	1.21 (0.92-1.37)
High blood pressure	0.71 (0.64-0.79)	1.08 (0.93-1.26)
High cholesterol	0.93 (0.83-1.04)	1.18 (1.02-1.37)
Osteoporosis	0.56 (0.44-0.72)	0.95 (0.65-1.39)

**Number of chronic diseases**		

None	1.00 (Referent)	1.00 (Referent)
One	1.00 (0.90-1.12)	1.26 (1.11-1.44)
Two or more	0.75 (0.67-0.84)	1.35 (1.16-1.56)

a Adjusted for sex, age, education, and income.
